# High density Huh7.5 cell hollow fiber bioreactor culture for high-yield production of hepatitis C virus and studies of antivirals

**DOI:** 10.1038/s41598-018-35010-5

**Published:** 2018-11-30

**Authors:** Anne F. Pihl, Anna F. Offersgaard, Christian K. Mathiesen, Jannick Prentoe, Ulrik Fahnøe, Henrik Krarup, Jens Bukh, Judith M. Gottwein

**Affiliations:** 1 Copenhagen Hepatitis C Program (CO-HEP), Department of Infectious Diseases, Hvidovre Hospital and Department of Immunology and Microbiology, Faculty of Health and Medical Sciences, University of Copenhagen, Copenhagen, Denmark; 2 Section of Molecular Diagnostics, Clinical Biochemistry, Aalborg University Hospital, Aalborg, Denmark

## Abstract

Chronic hepatitis C virus (HCV) infection poses a serious global public health burden. Despite the recent development of effective treatments there is a large unmet need for a prophylactic vaccine. Further, antiviral resistance might compromise treatment efficiency in the future. HCV cell culture systems are typically based on Huh7 and derived hepatoma cell lines cultured in monolayers. However, efficient high cell density culture systems for high-yield HCV production and studies of antivirals are lacking. We established a system based on Huh7.5 cells cultured in a hollow fiber bioreactor in the presence or absence of bovine serum. Using an adapted chimeric genotype 5a virus, we achieved peak HCV infectivity and RNA titers of 7.6 log_10_ FFU/mL and 10.4 log_10_ IU/mL, respectively. Bioreactor derived HCV showed high genetic stability, as well as buoyant density, sensitivity to neutralizing antibodies AR3A and AR4A, and dependency on HCV co-receptors CD81 and SR-BI comparable to that of HCV produced in monolayer cell cultures. Using the bioreactor platform, treatment with the NS5A inhibitor daclatasvir resulted in HCV escape mediated by the NS5A resistance substitution Y93H. In conclusion, we established an efficient high cell density HCV culture system with implications for studies of antivirals and vaccine development.

## Introduction

Hepatitis C virus (HCV) is an enveloped, positive-stranded RNA virus of the *Flaviviridae* family^1^. The single open reading frame (ORF) encodes a polyprotein of ~3000 amino acids (aa) that is cleaved into 10 proteins: Core, envelope glycoproteins E1 and E2, the viroporin p7, and the nonstructural (NS) proteins NS2, NS3, NS4A, NS4B, NS5A and NS5B^2–4^.

Each year 2 million new infections with HCV are estimated to occur worldwide. Approximately 80% of these individuals are not able to clear the infection and therefore develop chronic hepatitis^5,6^. Worldwide, 70–150 million individuals are estimated to be chronically infected^7–9^. Individuals with HCV-induced hepatitis typically show no or unspecific symptoms, but have an increased risk of developing liver cirrhosis and hepatocellular carcinoma. Thus, HCV is the leading cause of liver transplantations and is estimated to cause at least 400.000 deaths annually^8^.

Treatment with recently developed direct-acting antivirals (DAA) typically results in high cure rates^9–11^. However, only a fraction of infected individuals is treated, mostly because few infected individuals are aware of their status due to the lack of symptoms prior to the development of end-stage liver disease; further, because of the high cost of DAA^9^. In addition, evidence suggests that DAA treatment does not prevent reinfection and that for some patients treatment does not eliminate the risk of developing hepatocellular carcinoma following HCV eradication^12^. Finally, future efficacy of even the most efficient DAA regimens, including recently introduced pangenotypic regimens, will likely be compromised by the emergence and spread of resistant HCV variants^8,10,11,13^, as has been observed for other pathogens for which antimicrobials have been developed. Therefore, there is a large unmet need for a prophylactic HCV vaccine^13,14^.

To study HCV resistance to DAA and to develop a cell culture based HCV vaccine, cell culture systems are required^15^. All efficient infectious HCV cell culture systems employ the human hepatoma cell line Huh7 or derived cell lines, such as the Huh7.5 cell line, which are typically cultured in monolayers in cell culture flasks^16^. Initially, only a single HCV genotype 2a isolate (JFH1) could recapitulate the complete viral life cycle in cell culture^17,18^. Subsequently, various infectious cell culture systems producing HCV particles of the major genotypes were developed^15^. Of these systems, a JFH1-based recombinant with genotype 5a specific Core-NS2 with cell culture adaptive mutations showed the highest efficacy^19^. However, the described culture systems have several limitations. Cells grown in three-dimensional cultures might better resemble the *in vivo* environment^20,21^. Thus, for certain studies, such as studies of antivirals, a more physiological arrangement of cells than provided in monolayer cultures is considered beneficial^20–22^. In addition, virus yields in monolayer culture are typically limited, while development of a whole virus HCV vaccine and other applications, such as morphological studies of HCV particles, require large amounts of viral particles. However, no high-yield, high cell density HCV cell culture systems for efficient production of HCV have been established. Here we aim to establish a hollow fiber bioreactor platform for high cell density growth of the Huh7.5 cell line and the efficient production of HCV particles. Furthermore, we demonstrate the use of this platform for studies of DAA.

## Results

### Huh7.5 cell cultivation and HCV production in a hollow fiber bioreactor (HFBR)

**Figure 1:**
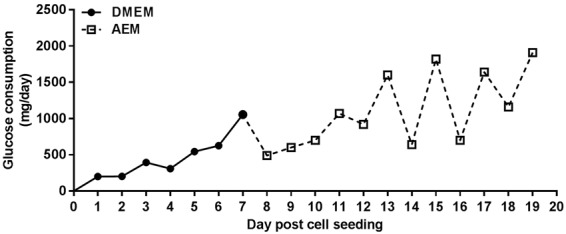
Cultivation of Huh7.5 cells in a hollow fiber bioreactor. 10^8^ Huh7.5 cells were seeded in a hollow fiber bioreactor in DMEM + 10% FBS. At day 7 post cell seeding, when glucose consumption was 1055 mg/day, DMEM was replaced with serum-free medium (AEM).

To establish high density cell culture with the Huh7.5 cell line, typically cultured in monolayer in cell culture flasks, we explored cultivation in a HFBR. Following cell seeding in serum-containing medium (DMEM + 10%FBS), glucose consumption gradually increased and reached ~1 g/day on day 7 post cell seeding (Fig. 1). From day 7, cultivation was continued in serum-free medium (AEM), as recommended for production of biological products in cell culture^23^. Glucose consumption decreased after media exchange to ~0.5 g/day on day 8 post cell seeding, but reached ~1 g/day on day 11 (Fig. 1).

**Figure 2:**
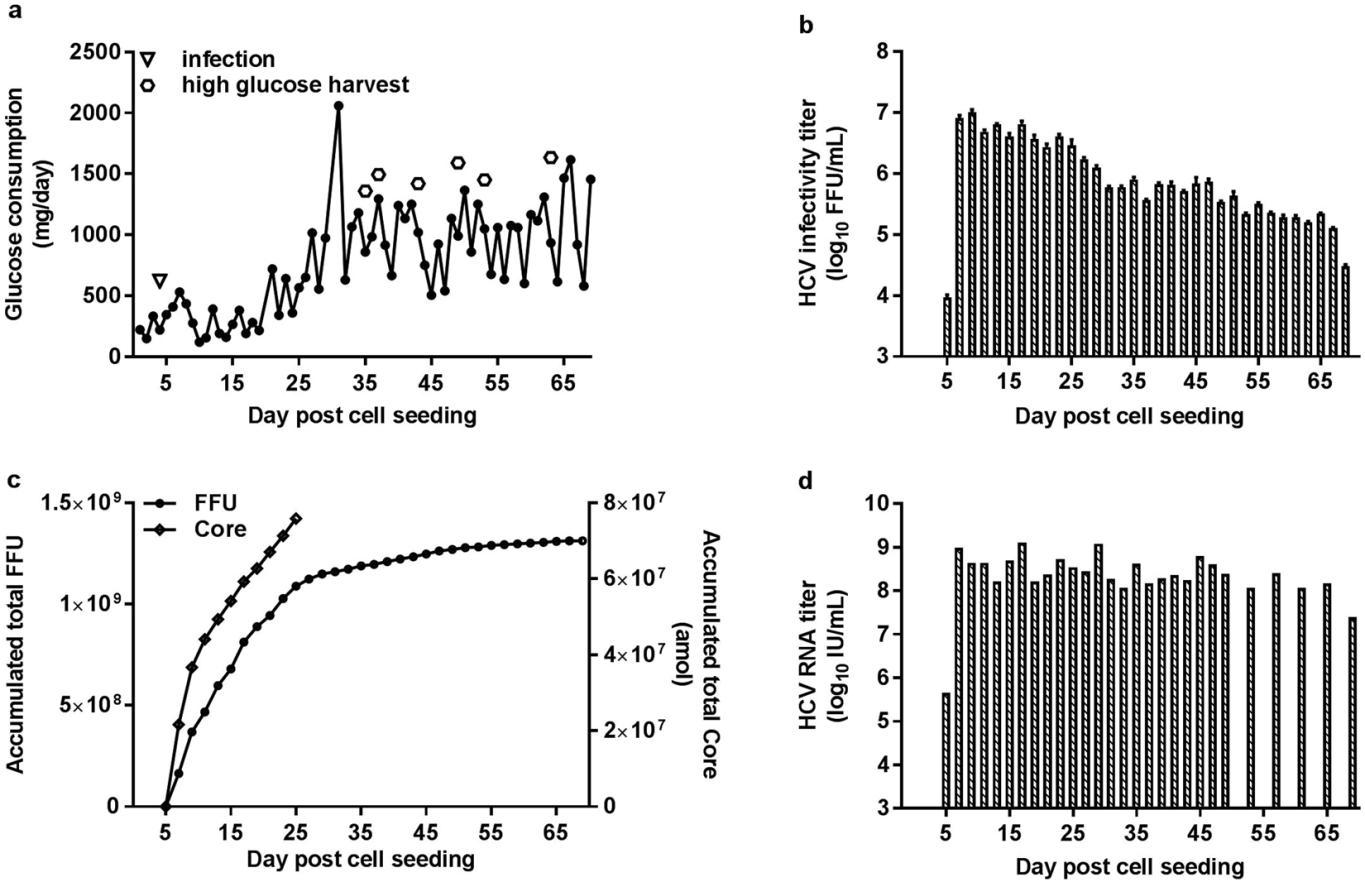
Production of HCV in a HFBR in DMEM + 10% FBS. (**a**) 10^8^ Huh7.5 cells were seeded in a HFBR and cultured in DMEM +10% FBS. On day 4 post cell seeding, when glucose consumption was 220 mg/day, cells were infected with 1.25 × 10^6^ FFU of HCV second passage stock. Arrow indicates time of infection; hexagons indicate high-glucose harvest. (**b**) Infectivity titers were determined as FFU/mL for each harvest collected following infection; shown are means of three replicates with error bars indicating standard error of the mean (SEM). (**c**) The total amount of FFU/HCV Core was determined by the addition of amounts of FFU/HCV Core from individual harvests. (**d**) HCV RNA titers were determined as IU/mL for selected harvests.

HCV production in HFBR was initially established under serum-containing conditions. In a second experiment, glucose consumption reached ~0.2 g/day on day 4 post cell seeding, when the cells were infected with 1.25 × 10^6^ FFU of HCV (Fig. 2a). Every second day approximately 20 mL supernatant were harvested from the HFBR extracapillary space. HCV infectivity titers of harvested supernatants increased soon after infection and peaked at 7.0 log_10_ FFU/mL on day 9 post cell seeding (day 5 post infection). Titers remained high for an extended period of time, but decreased to <6.0 log_10_ FFU/mL on day 31 following cell seeding and to <5.0 log_10_ FFU/mL on day 69, when the culture was terminated (Fig. 2b). The cumulative virus yield reached 1.09 × 10^9^ FFU at day 25 post cell seeding (day 21 post infection). Additional harvests only contributed slightly to the total amount of 1.31 × 10^9^ FFU, achieved when the experiment was terminated (Fig. 2c). The total amount of HCV Core accumulated during the main production phase (day 5–25 post cell seeding) was 0.76 × 10^8^ amol, amounting to ~1.6 μg Core (Fig. 2c). HCV RNA titers peaked at 9.1 log_10_ IU/mL on day 17 post cell seeding and declined to 7.4 log_10_ IU/mL at the end of cultivation (Fig. 2d). The mean specific infectivity of HFBR produced HCV was 1/133 FFU/IU (see Supplementary Fig. S1).

**Figure 3:**
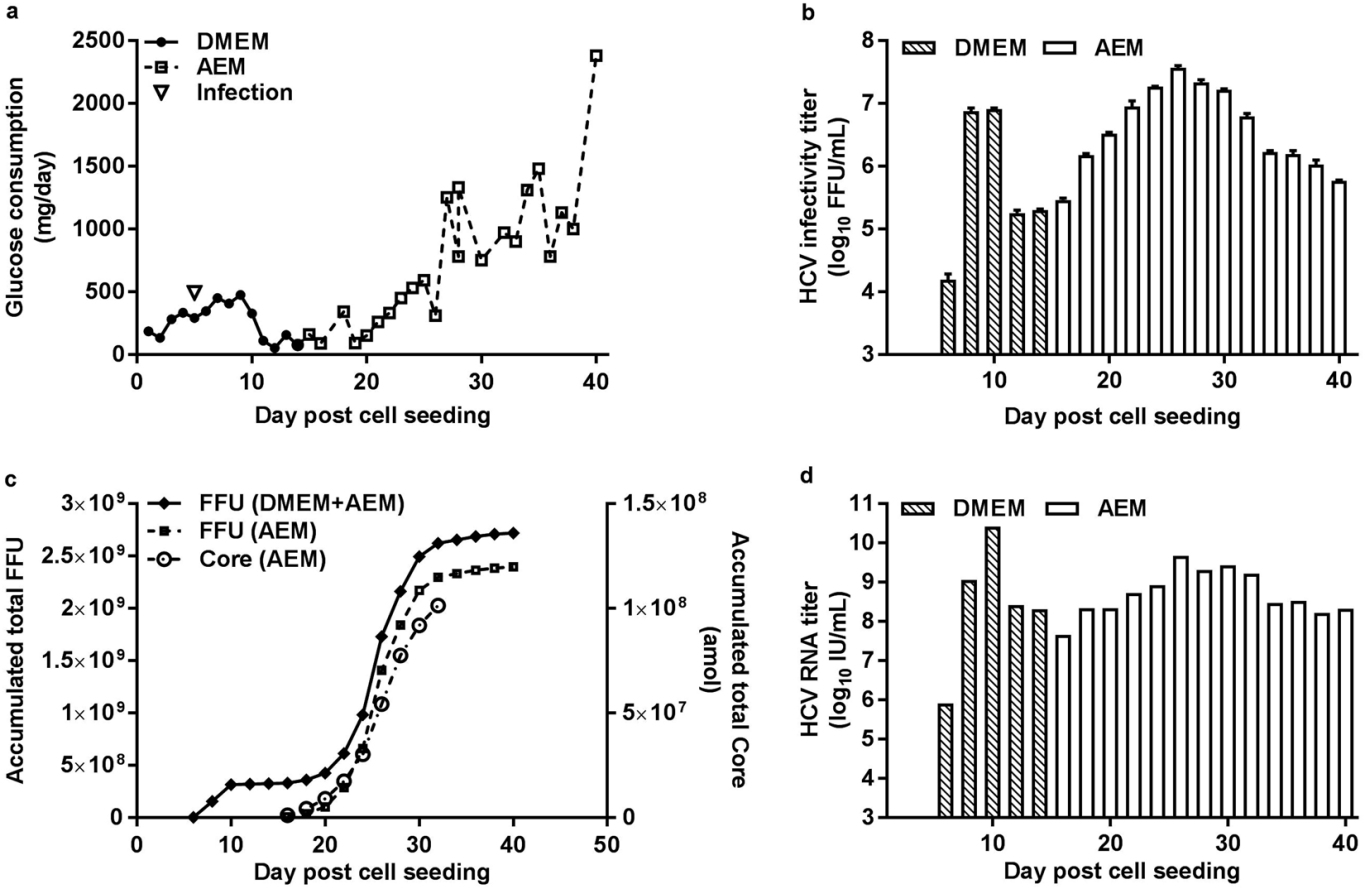
Production of HCV in a HFBR in serum-free AEM. (**a**) 1.8 × 10^8^ Huh7.5 cells were seeded in a hollow fiber bioreactor in DMEM + 10% FBS and infected with 1.25 × 10^6^ FFU of HCV second passage stock on day 5 post cell seeding (arrow), when glucose consumption was 290 mg/day. At day 14 post cell seeding DMEM was replaced with serum-free AEM. (**b**–**d**) Determination of HCV infectivity titers, accumulated FFU/HCV Core and RNA titers were carried out as described for Fig. 2.

To establish HCV production under serum-free conditions we carried out an experiment in which serum-containing DMEM was changed to serum-free AEM on day 14 post cell seeding (day 9 post infection) (Fig. 3a). A peak HCV infectivity titer of 7.6 log_10_ FFU/mL was recorded on day 26 post cell seeding; subsequently titers gradually decreased to 5.8 log_10_ FFU/mL on day 40, when the culture was terminated (Fig. 3b). A total amount of 2.7 × 10^9^ FFU were produced until day 40 post cell seeding (Fig. 3c). The largest quantity of 2.4 × 10^9^ FFU were produced in AEM, while 3.2 × 10^8^ FFU were produced in DMEM. The total amount of HCV Core accumulated during the main production phase in AEM (day 16–32 post cell seeding) was 1.0 × 10^8^ amol, amounting to ~2.1 μg Core (Fig. 3c). HCV RNA titers peaked at 10.4 log_10_ IU/mL on day 10 post cell seeding. The HCV RNA titers declined to 8.3 log_10_ IU/mL on day 40 at the end of the cultivation (Fig. 3d). The mean specific infectivity of HFBR produced HCV was 1/111 FFU/IU (see Supplementary Fig. S1).

### Long-term culture in HFBR did not result in further cell culture adaptation of HCV

**Figure 4:**
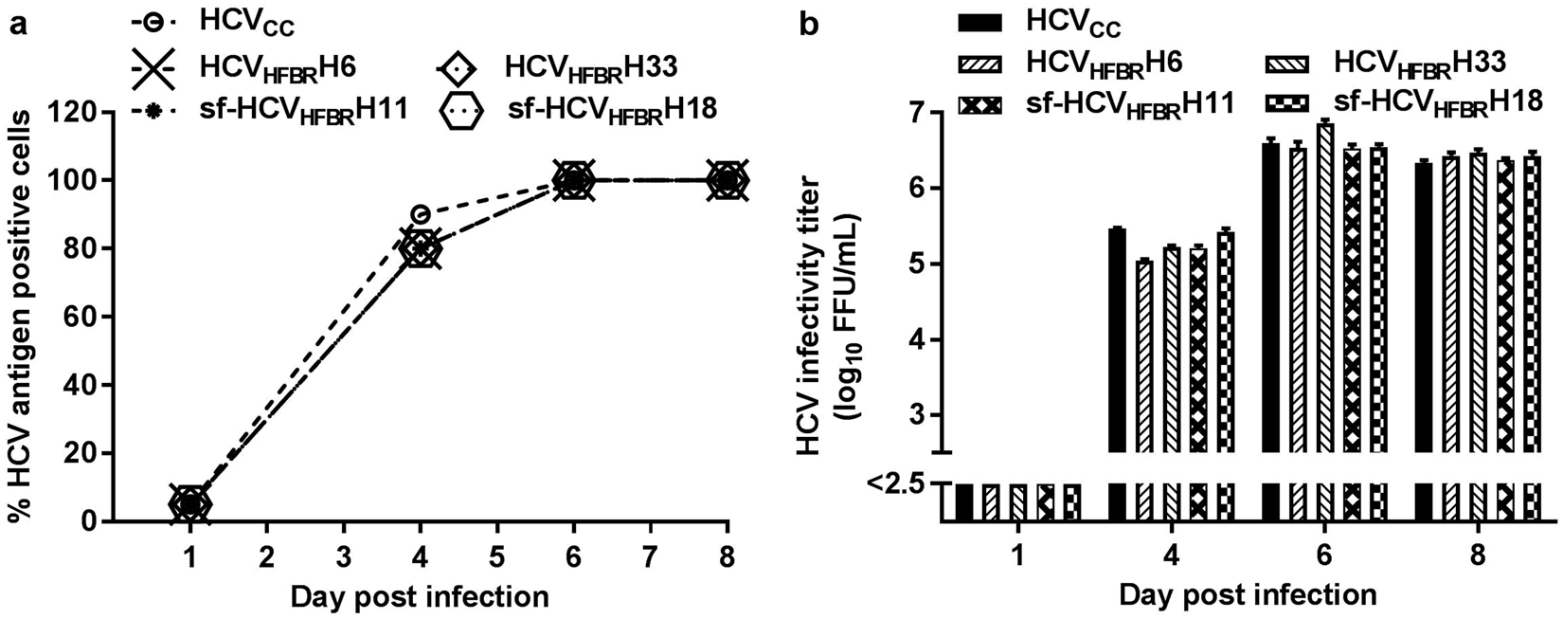
HFBR derived HCV show similar spread kinetics as standard monolayer cell culture derived HCV. Supernatant from harvest 6 and 33 of HFBR in DMEM + 10%FBS (HCV_HFBR_H6, HCV_HFBR_H33) (Fig. 2) and supernatant from harvest 11 and 18 of HFBR in serum-free AEM (sf-HCV_HFBR_H11, sf-HCV_HFBR_H18) (Fig. 3) were used to infect 10^6^ Huh7.5 cells plated the previous day in T25 cell culture flasks at an MOI of 0.003 in parallel with standard cell culture derived HCV third passage stock (HCVcc). Viral spread in the cultures was monitored by (**a**) determination of the % of HCV antigen positive cells based on immunostaining (**b**) determination of supernatant HCV infectivity titers. HCV infectivity titers are means of three replicates with standard error of the mean (SEM); the lower cutoff indicated by the y-axis break, was up to 2.5 log_10_ FFU/mL.

To evaluate if prolonged culture in HFBR resulted in further cell culture adaptation of HCV, we first compared viral spread characteristics and infectivity titers of HCV derived either from HFBR (HCV_HFBR_) or standard cell culture (HCV_cc_). HCV_HFBR_ and HCV_cc_ showed similar spread kinetics with viral spread to most culture cells on day 6 post infection (Fig. 4a), following infection of standard Huh7.5 cell cultures at MOI 0.003 with virus stocks specified in the Fig. 4 legend. Furthermore, HCV_HFBR_ and HCV_cc_ showed similar infectivity titers throughout the kinetic experiment (Fig. 4b). NGS analysis of the entire coding sequence revealed high genetic stability of HCV derived from early and late harvests from the bioreactors (Fig. 2 and 3) (see Supplementary Table S1). The ratio between coding and non-coding mutations (πN/πS), which ranged from 0.42 to 0.60, suggested negative selection. Further, pairwise distance analysis revealed a low level of heterogeneity of the viral population with only slight increases in heterogeneity over time (π < 10^−3^) (see Supplementary Table S1).

### HCV particles from HFBR have similar density as standard cell culture derived HCV

**Figure 5:**
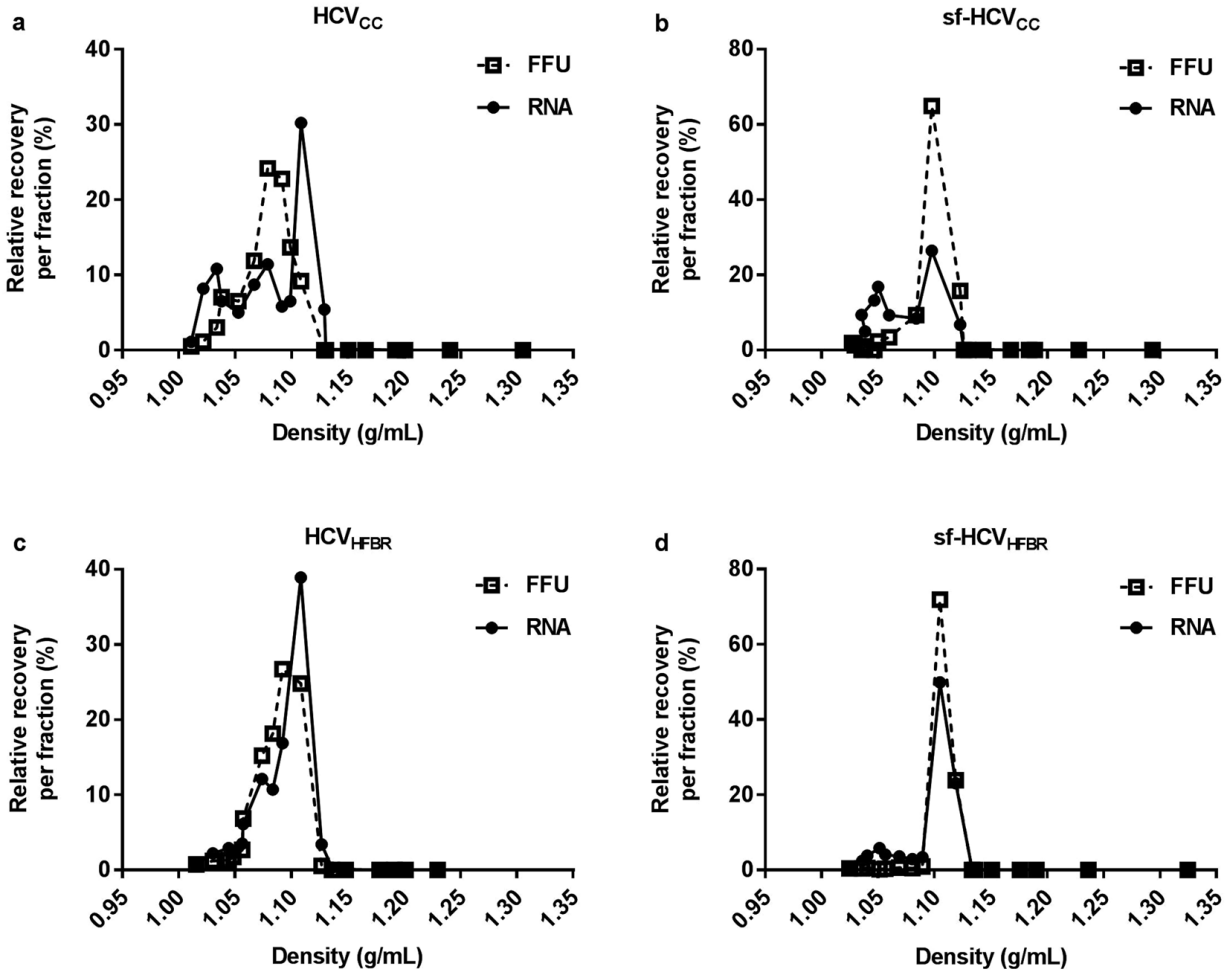
HFBR and standard monolayer cell culture derived HCV show similar density profiles. HCV particles used for density profiling on 10–40% iodixanol gradients were derived from: (**a**) standard cell culture in DMEM + 10%FBS using a third passage stock (HCVcc); (**b**) standard cell culture in serum-free AEM (sf-HCV_cc_); (**c**) harvest 6 of HFBR cultivation in DMEM + 10%FBS (HCV_HFBR_H6) (Fig. 2); or (**d**) harvest 11 of HFBR cultivation in serum-free AEM (sf-HCV_HFBR_H11) (Fig. 3). Following ultracentrifugation 18 fractions were collected, density, HCV infectivity and HCV RNA titers of each fraction were determined. Relative recovery of infectious virus and HCV RNA per fraction was calculated by relating the amounts detected in each fraction to the total amounts collected in all fractions.

As biophysical properties of HCV particles can depend on culture conditions^24^, we determined the buoyant densities of HCV derived from HFBR (Fig. 2 and 3) and from standard culture using equilibrium buoyant density ultracentrifugation on iodixanol gradients. In line with previous findings^24^, regardless of the cultivation method, viruses cultured in serum-containing medium showed a heterogeneous density profile (Fig. 5a and c), while viruses cultured in serum-free medium showed a homogeneous density profile (Fig. 5b and d). For sf-HCV_cc_ 81% of viruses were found in fractions with densities of 1.10–1.12 g/mL (Fig. 5b) and for sf-HCV_HFBR_ 96% of the viruses were found in these fractions (Fig. 5d). In contrast, these fractions contained only 23% of viruses for HCV_cc_ (Fig. 5a) and 25% of viruses for HCV_HFBR_ (Fig. 5c). In general, HCV RNA peaked with infectivity, suggesting an association with infectious HCV particles (Fig. 5a–d).

### HFBR derived HCV show similar sensitivity to neutralization with human monoclonal IgG and blocking of co-receptors CD81 and SR-BI as HCV from standard cell culture

**Figure 6:**
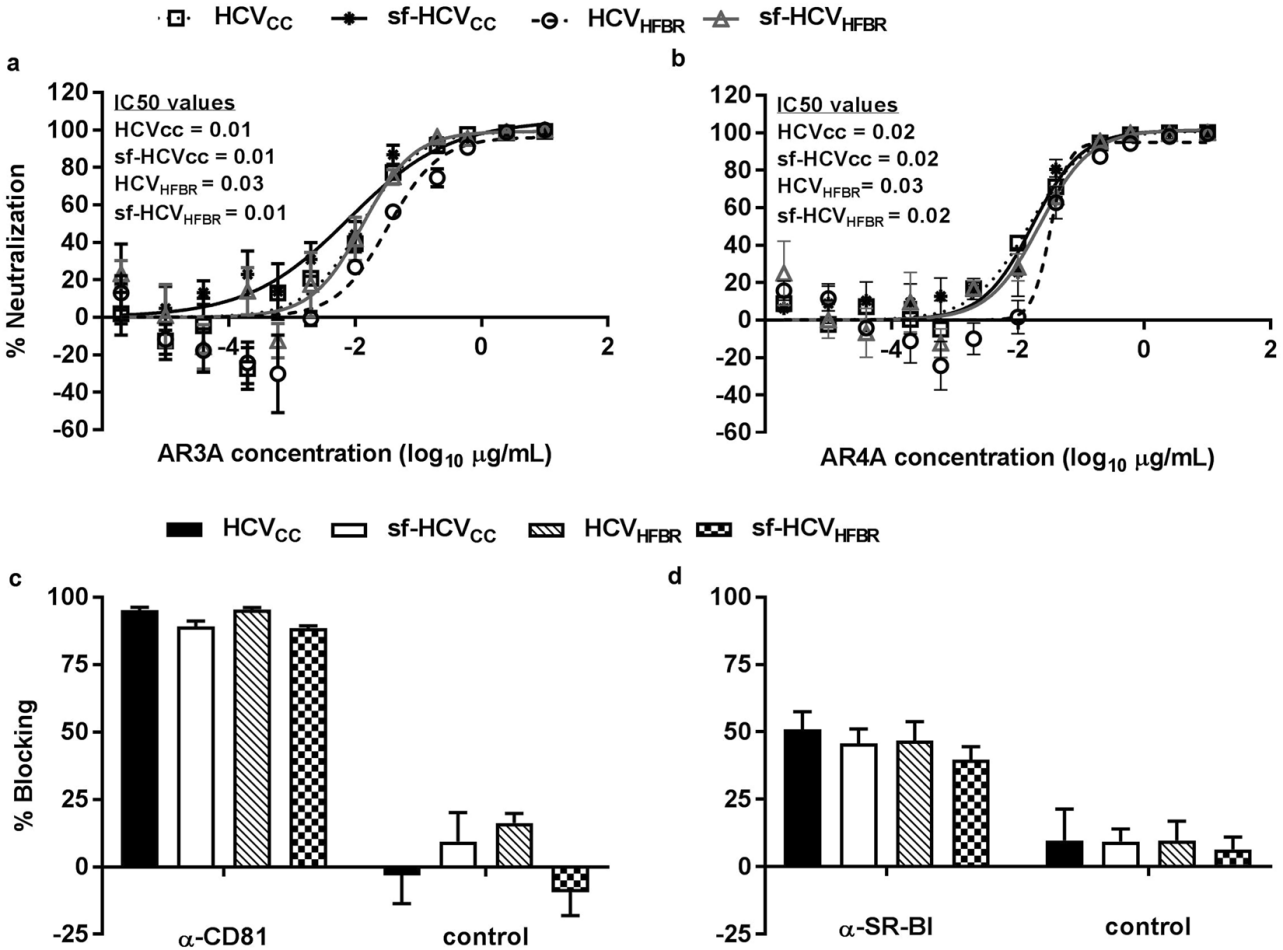
HFBR and standard monolayer cell culture derived HCV show similar sensitivity to monoclonal HCV neutralizing antibodies and similar dependency on entry co-receptors CD81 and SR-BI. Neutralization assays with human monoclonal antibodies (**a**) AR3A and (**b**) AR4A and receptor blocking assay with (**c**) anti-CD81 and (**d**) anti-SR-BI were carried out as described in Materials and Methods. HCV was from (i) a third passage stock generated in standard cell culture in DMEM + 10%FBS (HCVcc); (ii) a forth passage stock generated in standard cell culture in serum-free AEM (sf-HCV_cc_); (iii) HFBR cultivation in DMEM + 10%FBS (HCV_HFBR_H6) (Fig. 2); or (iv) HFBR cultivation in serum-free AEM (sf-HCV_HFBR_H11) (Fig. 3). (**a** and **b**) The % neutralization was calculated and curves were fitted as described in Materials and Methods. Data points are means of three replicates with SEM. IC50 values are shown (μg/mL). (**c** and **d**) The % blocking was calculated as described in Materials and Methods. Data points are means of eight replicates with SEM.

To investigate whether the cultivation in the bioreactors had an influence on viral characteristics mediated by the HCV envelope proteins, we carried out neutralization and receptor blocking assays. We first determined HCV sensitivity to well-defined human monoclonal antibodies AR3A (Fig. 6a) that targets a conformational epitope in E2 and AR4A (Fig. 6b) that targets a conformational epitope in E1E2^25,26^. HCV_cc_, sf-HCV_cc_, HCV_HFBR_ and sf-HCV_HFBR_ showed similar sensitivity to both monoclonal antibodies with IC50 values ranging from 0.01 to 0.03 μg/mL. We also tested if the requirement for entry co-receptors CD81 (Fig. 6c) and SR-BI (Fig. 6d) to establish infection was altered in HCV derived from the bioreactors. HCV_cc_, sf-HCV_cc_, HCV_HFBR_ and sf-HCV_HFBR_ showed similar dependency on co-receptor CD81 as antibody-mediated blocking of co-receptor CD81 resulted in 88–95% blocking of infection. HCV grown on different platforms and under different conditions also showed similar dependency on co-receptor SR-BI with antibody-mediated blocking of co-receptor SR-BI resulting in 40–51% blocking of infection.

### HCV culture in HFBR as model system for studies of antiviral resistance

**Figure 7:**
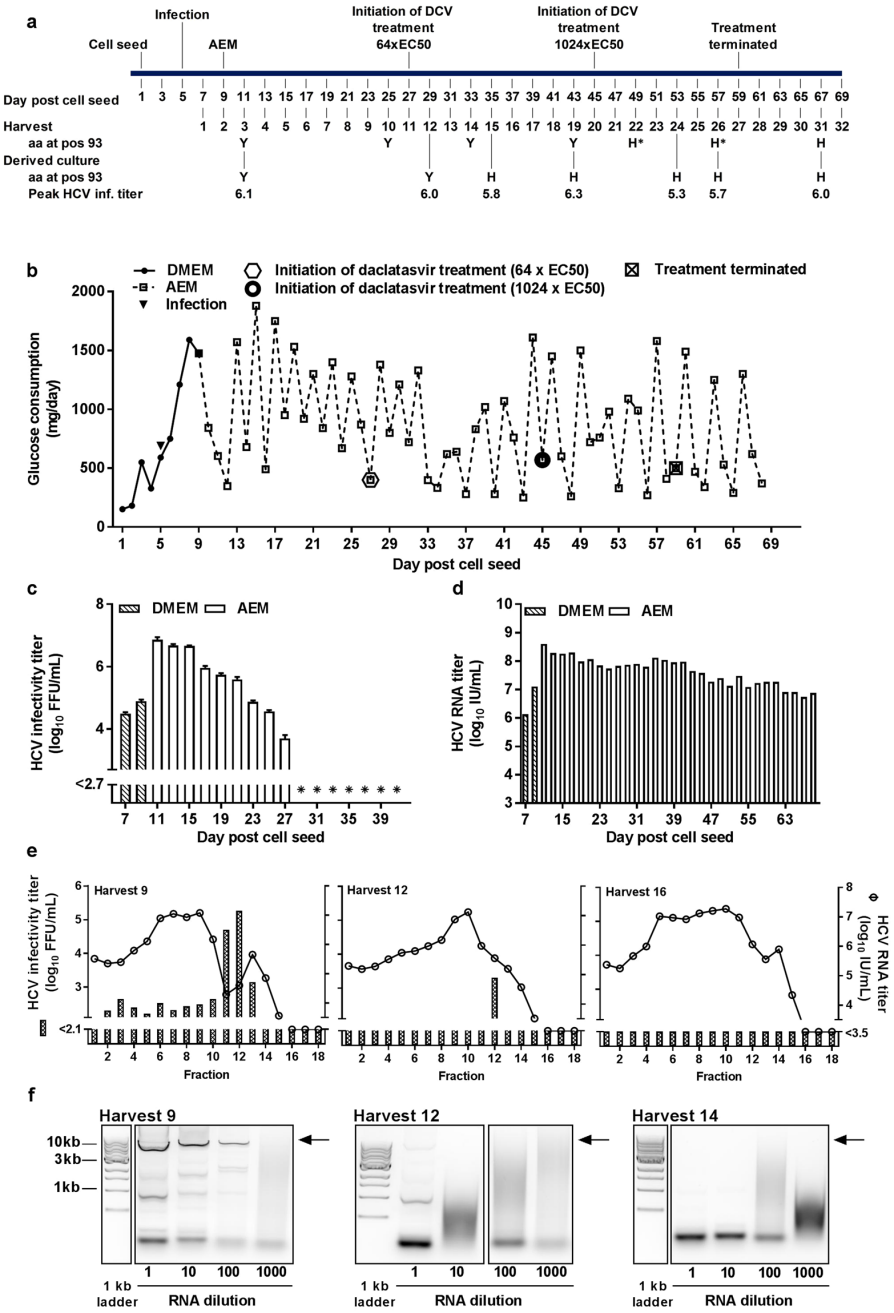
Induction of HCV escape from a direct acting antiviral in HFBR. (**a**) Timeline of Huh7.5 cell cultivation in DMEM + 10%FBS and serum-free AEM, indicating infection with HCV and initiation of treatment with different concentrations of the NS5A inhibitor daclatasvir (DCV). HCV from HFBR harvests (1000 μL, 50 μL or 5 μL) were used for infection of 10^6^ Huh7.5 cells plated the previous day in T25 cell culture flasks (derived cultures). Peak infectivity titers in derived cultures are reported. Amino acid at NS5A position 93 according to the H77 reference sequence (GenBank accession no AF009606) was determined by Sanger sequencing for selected harvests and derived cultures. *, H at position 93 was present in ~50% of viral genomes. (**b**) 8 × 10^7^ Huh7.5 cells were seeded in a hollow fiber bioreactor in DMEM + 10% FBS and infected with 1.25 × 10^6^ FFU of HCV third passage stock on day 5 post cell seeding, when glucose consumption was 590 mg/day (arrow). At day 9 post cell seeding DMEM was replaced with serum-free AEM. Treatment with daclatasvir at 7.8 nM (corresponding to 64 x EC50^29^) was initiated at day 27 post cell seeding; at day 45 the concentration was increased to 124.2 nM (corresponding to 1024 x EC50). On day 59 the treatment was terminated. (**c** and **d**) Determination of HCV infectivity titers and HCV RNA titers were carried out as described in Fig. 2. *, indicates HCV infectivity titers below assay detection level. (**e**) HCV from harvest 9, 12 and 16 were loaded on 10–40% iodixanol gradients. Following ultracentrifugation 18 fractions were collected as in Fig. 5. HCV infectivity and HCV RNA titers of each fraction were determined as described for Fig. 2. (**f**) RNA extracted from harvest 9, 12 and 14 at 1-, 10-, 100- and 1000-fold dilution was used for RT-PCR for generation of a full-length amplicon spanning the complete HCV ORF. PCR products were visualized on a 1% agarose gel including a 1 kb DNA ladder; expected positions of full-length amplicons are indicated by arrows. Gel image was cropped as indicated by boxes; full-length gel is presented in Supplementary Fig. S4.

Cell densities in tissues are better mimicked by HFBR than monolayer cell cultures, making HFBR relevant platforms for studies of antivirals^27,28^. We previously studied HCV escape from antivirals in standard cell culture^29–33^, and in this study we set out to investigate if the HFBR could be used as a platform to study HCV escape from the DAA daclatasvir targeting HCV NS5A^34^. In a HFBR, cells were infected with 1.25 × 10^6^ FFU of HCV at day 5 post cell seeding when glucose consumption was ~0.6 g/day (Fig. 7a and b). On day 9 post cell seeding (day 4 post infection) serum-containing medium was exchanged for serum-free AEM. HCV infectivity and HCV RNA titers of the harvests increased after infection and peaked at 6.9 log_10_ FFU/mL and 8.6 log_10_ IU/mL on day 11 post cell seeding (Fig. 7c and d). On day 27 post cell seeding daclatasvir was added to the medium at a concentration corresponding to 64xEC50 of HCV recombinants with NS5A of isolate JFH1^29^ and on day 45 post cell seeding the daclatasvir concentration was increased to 1024xEC50 (Fig. 7a and b). Following initiation of daclatasvir treatment it was no longer possible to determine HCV infectivity titers (Fig. 7c). To investigate if infectious viruses were present in the bioreactor under treatment, we used harvest supernatants to infect cells in standard cell cultures designated derived cultures. All derived cultures became infected and showed peak titers of ~6 log_10_ FFU/mL. Direct sequence analysis of daclatasvir-treated HFBR derived HCV revealed presence of NS5A inhibitor resistance associated substitution Y93H in approximately 50% of the viral population at harvest 22 and 26, whereas most genomes were estimated to carry this substitution at harvest 31 (Fig. 7a and Supplementary Table S2). To confirm that Y93H in HFBR derived HCV conferred resistance to daclatasvir, we carried out a short-term treatment assay (see Supplementary Fig. S2). For HCV derived from the HFBR prior to treatment EC50 of daclatasvir was 0.11 nM, while for HCV derived from the HFBR during treatment EC50 of daclatasvir was 291 nM. Thus, HFBR derived HCV escape variants showed 2645-fold resistance, in accordance with fold resistance values previously observed for JFH1 with Y93H^29,35^.

Given that HCV NS5A inhibitors interfere with HCV RNA replication^2^ and given that we previously had observed a decrease in HCV RNA titers during treatment with the NS5A inhibitor velpatasvir and the NS5B inhibitor sofosbuvir in standard monolayer cell cultures^30,31^, it was an unexpected finding that HCV RNA titers did not decrease during daclatasvir treatment in the bioreactor (Fig. 7d). To ensure the efficacy of daclatasvir treatment and to facilitate determination of HCV infectivity titers during treatment, we carried out iodixanol gradient ultracentrifugation of selected bioreactor harvests obtained prior to and following initiation of treatment. We confirmed that daclatasvir treatment resulted in a strong decrease in the total amount of infectious HCV detected across all ultracentrifugation fractions: 2.4 × 10^5^ FFU were detected in harvest 9 prior to treatment, but only 2.1 × 10^3^ FFU in harvest 12 on day 2 following initiation of treatment. In harvest 16 on day 10 following initiation of treatment, no infectious viruses were detected in any of the analyzed fractions (Fig. 7e). While total amounts of HCV RNA measured by TaqMan real time PCR detected across all ultracentrifugation fractions were similar before and after initiation of treatment (Fig. 7e), we observed a >100-fold decrease in full-length HCV RNA following treatment initiation (Fig. 7f). Finally, we confirmed that in standard monolayer cell culture daclatasvir treatment resulted in a decrease in HCV RNA titers, which was similar to that observed under sofosbuvir treatment (see Supplementary Fig. S3). Thus, in the bioreactors daclatasvir treatment resulted in a decrease in infectious viruses and full-length viral RNA. However, in contrast to standard cell culture, in the bioreactor treatment did not have a significant impact on HCV RNA titers determined by TaqMan PCR using short amplicons.

## Discussion

We provide proof-of-concept for high cell density culture of the highly HCV permissive Huh7.5 hepatoma cell line in a hollow fiber bioreactor. We showed that this platform can be used to produce high titer HCV stocks, with bioreactor derived HCV showing similar characteristics as HCV derived from standard monolayer cell culture. In addition, we demonstrated the use of this platform for studies of HCV antivirals.

High density mammalian cell culture is required for various applications including production of pharmaceutical proteins, monoclonal antibodies, and vaccine antigens^36–38^. Most licensed antiviral vaccines employ whole viral particles as antigens^39,40^. For several of these vaccines, including polio, rabies, and Japanese encephalitis virus vaccines, production platforms have been optimized using classical cell lines such as Vero cells, which are typically grown in stirred tank bioreactors on microcarrier beads^20,41^. However, production of certain other viruses is not supported by established cell lines and culture conditions. For example, cell lines required for production of certain viruses grow adherent to surfaces and might be easier to culture in bioreactors inducing less shear stress than stirred tank bioreactors^37,38^.

Efficient production of HCV in cell culture is limited to adherent Huh7 derived cell lines, such as Huh7.5 cells^15,16^. Establishment of high density cultures of these cells to enable high-yield production of HCV is of great importance for the development of a whole HCV particle vaccine^42–44^, but also for other applications, such as studies of HCV morphology, and studies that require infection at high MOI. To our knowledge there are only two reports on high density Huh7 cell culture for production of HCV in bioreactors. Murakami *et al*. describe cultivation of Huh7 cells expressing a full-length genotype 1b HCV recombinant on carrier beads in a radial-flow bioreactor, which only resulted in limited production of HCV particles^45^. Sainz *et al*. cultivated Huh7 cells on microcarrier beads using a rotating wall vessel bioreactor^46^. When infected with the HCV genotype 2a recombinant JFH1, this platform yielded HCV infectivity titers of 4–5 log_10_ FFU/mL.

In the bioreactor used here, peak HCV infectivity titers of up to 7.6 log_10_ FFU/mL were achieved using a JFH1-based recombinant with genotype 5a specific Core-NS2. These titers were considerably higher than observed for the same virus in monolayer cell culture^19^, and higher than titers observed by Murakami *et al*. and Sainz *et al*.^45,46^, as well as titers observed for cell culture derived HCV in general. This is likely due to concentration of the virus by retention in the bioreactor cartridge, which is separated from the media supply. Also, media is supplied in perfusion mode, providing efficient exchange of nutrients, metabolites and oxygen^47^. We excluded that the high HCV infectivity titers observed were due to an increase in HCV fitness during high cell density culture, since monolayer cultures infected with bioreactor derived HCV did not yield increased infectivity titers. In addition, next generation sequence analysis of HCV harvested from the bioreactor showed high genetic stability without indications of significant adaptation to high density culture conditions. In line with previous observations in monolayer cell cultures, we observed higher HCV infectivity titers in serum-free than in serum-containing high density cell culture^24^. During the main production phase in serum-free medium ~2 μg HCV Core were produced in one bioreactor. Assuming a recovery of ~50% during downstream processing required for purification and concentration of HCV antigen, one bioreactor will eventually yield ~1 μg purified HCV Core antigen. This is in the range of what would be used in mouse immunogenicity studies for one immunization of one animal. Of note, also human antiviral vaccines, such as the TBE vaccine, operate in this range. However, even though a larger model of the described bioreactor with a 4-fold greater surface area is available, high density Huh7.5 cell cultures in bioreactors allowing scalability to industrial scale are likely to be required for development of a whole HCV particle vaccine.

For development of a whole virus particle vaccine for human use, virus production under serum-free conditions is required^23^. In contrast to the previous reports on Huh7 cell based production of HCV in bioreactors^45,46^, we established high cell density production of HCV under serum-free conditions based on protocols previously developed in monolayer Huh7.5 cell culture^24^. As previously reported for HCV produced in monolayer serum-free cell culture, HCV produced in high cell density serum-free culture in the bioreactor also showed a homogeneous density profile. A homogeneous density profile is of great advantage for downstream purification processes relying on density-based separation, for example ultracentrifugation. In contrast to previous observations in a small-scale three-dimensional Huh7 cell culture model^48^, high density culture did not change the HCV density profile, suggesting that lipoprotein association was not altered compared to HCV derived from monolayer cell culture.

Further phenotypic characterization of bioreactor derived HCV showed that high cell density culture did not result in altered viral dependency on key HCV entry receptors, which is in line with previous findings^48^. In addition, HCV derived from high cell density and monolayer cell culture showed similar neutralization profiles indicating that important conformational neutralization epitopes on the envelope proteins showed similar accessibility to neutralizing antibodies, which is of importance for vaccine development. The specific infectivity of bioreactor produced HCV was slightly lower than that of HCV produced in monolayer cell culture (see Supplementary Fig. S1), which might be due to delayed clearance of non-full-length RNA from the bioreactor (also see discussion below).

In future studies, it would be of interest to use the developed platform to investigate the influence of HCV infection on the cellular metabolism such as glucose consumption.

Three-dimensional cell culture systems are used for various applications, and especially for studies of drug pharmaceuticals including antivirals. Thus, the hollow fiber bioreactor used in this study has previously been used for studies of antivirals^22,49^. While three-dimensional cell cultures have been developed for various cell types, liver cell systems are of special interest given the important role of the liver in drug metabolism and different three-dimensional liver cell models have been developed^20,21,47,50–52^. Various efforts have been undertaken to culture Huh7 cells in a three-dimensional arrangement^52–57^. However, only a limited number of studies investigated HCV infection in such cultures. Thus, in addition to the two bioreactor-based systems described above^45,46^, different small-scale systems have been developed^45,48,58,59^. Of these, only one system using Huh7.5 cells showed reasonable HCV production with infectivity titers of 4–5 log_10_ FFU/mL^58^. It should be noted that a smaller version of the bioreactor used here with a 7-fold smaller surface area is available, which could facilitate more extended analysis of antivirals and cell metabolism in future studies.

We obtained proof-of-concept that the established high density culture platform could be used to study HCV escape from DAA. Here, HCV escape occurred after a similar treatment interval as in a previous study in monolayer cell cultures using the same NS5A inhibitor and an HCV recombinant with NS5A of the same isolate (JFH1)^29^. We previously found that not only viruses with the escape substitution Y93H, but also viruses with F28S were selected in daclatasvir treated monolayer cultures, whereas only viruses with Y93H were found in the bioreactor. Both substitutions confer high resistance to daclatasvir without obvious impairment of HCV fitness *in vitro* and are also found for HCV genotype 2 infected patients with treatment failure *in vivo*^11,29^. In the bioreactor, daclatasvir treatment resulted in a decrease in infectious viruses and full-length HCV RNA. However, in contrast to findings in monolayer cell culture^30,31^ (see Supplementary Fig. S3), treatment did not decrease HCV RNA titers determined by TaqMan real time PCR, which is based on a small amplicon. This might be due to relatively inefficient clearance of non-full-length HCV RNA from the bioreactor during harvests, caused by differences in handling compared to monolayer cell cultures: Thus, during harvests in monolayer cell culture, supernatant is removed completely, cells are washed with PBS and in serum-containing cultures also trypsinized before fresh medium is added. However, in the bioreactor supernatant is merely flushed out while replaced by fresh medium without cell washing and with a significant dead space. This hypothesis is further supported by the finding that Y93H was detected earlier in derived monolayer cultures infected with viable viruses showing resistance to daclatasvir than in the bioreactor, where apparently RNA fragments of non-viable viruses without resistance to daclatasvir persisted. Finally, also in non-treated bioreactors (Fig. 2 and 3) over time infectivity declined faster than HCV RNA levels determined by TaqMan real time PCR.

Importantly, Huh7.5 cells allow not only growth of HCV but also various other important and emerging human pathogenic viruses such as Zika, chikungunya, dengue, West Nile and yellow fever virus, for which vaccines or antivirals remain to be developed^60–64^.

In conclusion, we established high-yield production of HCV in high cell density human hepatoma cell culture, which might be an important step towards development of a whole HCV particle vaccine.

## Materials and Methods

### Standard monolayer cultivation of Huh7.5 cells

For expansion of cells for seeding in HFBR and for other experiments in standard cell culture, human hepatoma Huh7.5 cells^16^ were cultured at 37 °C and 5% CO_2_ in flasks in monolayers in Dulbecco’s Modified Medium (DMEM) with 10% fetal bovine serum (FBS) as described^65^. All culture media were supplemented with 100 U/mL penicillin +100 μg/mL streptomycin. Cells were split every 2–3 days. HCV infection was carried out by inoculation with virus containing supernatant as specified followed by overnight incubation. Selected cultures were treated with daclatasvir or sofosbuvir (Acme Biosciences) as specified.

### Standard monolayer cell culture derived HCV virus stocks

The HCV recombinant SA13/JFH1_Core-NS5B_ containing Core, E1, E2, p7 and NS2 of isolate SA13 (genotype 5a), and NS3, NS4A, NS4B, NS5A, NS5B and UTRs of isolate JFH1 (genotype 2a) was used and is referred to as HCV^19^. Throughout the study three virus stocks were used: (i) a previously described second passage virus stock grown in DMEM + 10%FBS (HCVcc)^19^; (ii) a derived third passage virus stock grown in DMEM + 10% FBS (HCVcc); (iii) a derived fourth passage virus stock grown in Adenovirus Expression Medium (AEM) without serum (LifeTechnologies) (sf-HCVcc) as described^24^. For all virus stocks, HCV genetic stability was confirmed by Sanger sequencing of the entire ORF. For each experiment the identity of the used stock is indicated in the figure legend.

### Cultivation of Huh7.5 cells in HFBR

The HFBR cartridge C2011 (FiberCell Systems) was pre-cultured in phosphate buffered saline (PBS) for 48 hours, DMEM for 24 hours, and DMEM + 10%FBS for 24 hours by perfusion of the intracapillary space. Huh7.5 cells cultivated under standard conditions were pelleted and resuspended in ~20 mL conditioned media collected prior to cell harvesting. Cells were seeded in the extracapillary space through the left side port. The HFBR was supplied with 125 mL DMEM + 10%FBS, administered into the intracapillary space, and maintained at 37 °C and 5% CO_2_. Glucose concentration of the media supply was measured daily using an AccuCheck device (Roche). Volume of the media supply was doubled when glucose concentration was <2 g/L until a maximal volume of 500 mL. Media flow in the HFBR was controlled by a peristaltic pump (FiberCell® Systems Duet Pump, # P3202), initially at ~80 mL/min and after 3–4 days at ~110 mL/min. HCV infection was carried out as specified by injecting HCV in a total volume of ~20 mL DMEM + 10%FBS into the extracapillary space. Serum-free culture was initiated by replacing the media in the extracapillary space with ~20 mL and the media supply with 1 L serum-free AEM. High glucose harvest was carried out when glucose consumption repeatedly was >1 g/day by flushing media and cells in the extracapillary space back and forth using both side ports, ultimately removing 20 mL cell suspension. For the treated HFBR, daclatasvir (Acme Bioscience) was diluted in dimethyl sulfoxide and added to the media supply at the specified concentrations.

### Evaluation of HCV infected cultures

HCV spread in standard monolayer cell culture was evaluated by immunostaining using primary antibody anti-NS5A-9E10 at 1:3000^17^, secondary antibody Alexa Flour® 488 goat anti-mouse IgG at 1:500 (Lifetechologies) and Hoechst 33342 at 1:1000 as described^24,65^. The percentage of infected cells was determined by fluorescence with a Zeiss Axio Vert.A1 microscope.

To determine HCV infectivity titers, Huh7.5 cells were plated at 6000 cells/well in poly-D-lysine 96-well plates (Thermo Scientific). The next day, serially diluted viral supernatant was added (minimal dilution was 2-fold), testing each dilution in triplicates, and incubated for 48 hours^24,66^. Cells were immunostained as described using anti-NS5A-9E10 at 1:5000^17^ and ECL sheep anti-mouse IgG horseradish-peroxidase linked whole antibody (GE Healthcare) at 1:500, followed by incubation with DAB substrate (DAKO). FFU were counted automatically with an ImmunoSpot Series 5 UV Analyzer (CTL Europe Gmbh) with customized software. Titers were calculated as described^35,67^.

HCV Core titers in culture supernatant were determined using the ARCHITECT HCV Ag assay (Abbott).

HCV RNA titers were determined from RNA extracted from 200 μL pre-diluted supernatant using the Total Nucleic Acid Isolation Kit (Roche Applied Science) and by Taqman real-time PCR as described^65^.

### Equilibrium density gradient ultracentrifugation

Ultracentrifugation was done as described^68^. In brief, OptiPrep™ Density Gradient Medium (Sigma) was used to make 10%, 20%, 30% and 40% iodixanol dilutions in PBS. Dilutions were layered on top of each other using 2.5 mL per dilution in a Beckman centrifuge tube (Ramcon) and incubated at 4 °C. The following day, 1–2 mL of viral supernatant was loaded on top of the gradients, tubes were loaded in a Beckman SW-41 rotor and spun at 35,000 rpm for 18 hours at 4 °C in a Beckman XL-70 Ultracentrifuge. Following centrifugation, 18 fractions of ~550 μL were collected from the bottom of each vial and 400 μL of each fraction were weighed to determine the density^68^. The HCV infectivity titer of each fraction was determined as described above.

### Sequence analysis

For Sanger sequence analysis of NS5A Domain I, HCV RNA was extracted from 200 μL supernatant with High Pure Viral Nucleic Acid Kit (Roche). Superscript III reverse transcriptase (Thermo Fisher Scientific) was used for first-strand cDNA synthesis and Advantage 2 Polymerase Mix (Takara Bio) for PCR amplification as described^65^ (for specific primers and protocols see Supplementary Materials and Methods including Supplementary Tables S3, S4, S5). Obtained amplicons were subjected to Sanger sequencing (Macrogen Europe).

Overall, sequence analysis of the complete ORF was done as described^31^. In brief, HCV RNA was extracted from 250 μL supernatant with TRIzol LS and chlorofrom in Gel Lock heavy Eppendorf tubes and purified on RNA Clean & Concentrator ™-5 columns (Zymo Research). Maxima H Minus Reverse Transcriptase (ThermoScientific) was used for first-strand cDNA synthesis and Q5® Hot start High-Fidelity DNA Polymerase (New England Biolabs) was used for PCR amplification (for specific primers and protocols see Supplementary Materials and Methods including Supplementary Tables S6, S7, S8)^31^. PCR samples were purified with DNA clean & concentrator ™-25 (Zymo Research) and used for Sanger sequencing or next generation sequence analysis (NGS). For NGS, the resulting PCR product was in addition purified by extraction from a 1% TAE gel using Zymoclean large fragment DNA recovery kit (Zymo Research). Library preparation was done by Macrogen Inc by Truseq Nano or in-house by NEBnext Ultra II DNA Library Prep Kit. Sequence analysis was carried out on the Illumina Miseq platform and DNA analysis was performed to determine single nucleotide polymorphism variants and genetic stability as described previously^69,70^.

### HCV neutralization

Huh7.5 cells were plated at 6000 cells/well in poly-D-lysine 96-well plates. The next day, monoclonal antibodies AR3A or AR4A^25,26^ were serially diluted in DMEM + 10%FBS. Fifty µL of each dilution were mixed with an equal volume of virus in triplicates and incubated for 1 hour at 37 °C in parallel with 12 replicates of virus only controls. Mixtures were added to the cells and incubated for 3 hours at 37 °C before washing with pre-warmed PBS. Fresh media were added followed by incubation for 48 hours at 37 °C. Cells were immunostained and single HCV positive cells were counted as described for HCV infectivity titrations^35,71^. The percent neutralization was calculated relating the number of single HCV-positive stained cells in wells infected with antibody-treated virus to the mean number of stained cells in wells with virus only controls. Antibody concentrations were logarithmically transformed, sigmoidal dose-response curves were fitted $y=top/(1+{10}^{(log10EC50-X)\times hillslope})$ and IC50 values were calculated using Graphpad Prism 7.0.

### HCV receptor blocking

Huh7.5 cells were plated at 6000 cells/well in poly-D-lysine 96-well plates. The next day eight replicate cultures were incubated with either 10 µg/mL of monoclonal antibody against CD81 (BD Pharmingen, JS81) or the control antibody 553447; or against SR-BI (C16-71) or the control antibody D^72^, diluted in DMEM + 10% FBS, while eight replicate cultures served as virus only controls. Following 1 hour incubation at 37 °C, virus containing supernatants were added followed by incubation for 4 hours at 37 °C before washing with pre-warmed PBS. Fresh media were added followed by incubation for 48 hours at 37 °C. Cells were immunostained and FFU were counted as described for infectivity titrations. The percent blocking was calculated relating the number of FFU in antibody-treated wells to the mean number of FFU in untreated wells. Data points are means of eight replicates with SEM.

### Treatment assay

Huh7.5 cells were plated at 6000 cells/well in poly-D-lysine 96-well plates. The next day, virus containing supernatants were used for infection. The following day, daclatasvir was serially diluted in DMEM+10%FBS and added to the cells; each concentration was tested in triplicates. After 48 hours, cells were immunostained as described for HCV infectivity titrations and single HCV positive cells were counted as described for infectivity titrations. The percent inhibition was calculated relating the number of single HCV-positive stained cells in treated wells to the mean number of stained cells in untreated wells. Daclatasvir concentrations were logarithmically transformed, sigmoidal dose-response curves were fitted $y=top/(1+{10}^{(log10EC50-X)\times hillslope})$ and IC50 values were calculated using Graphpad Prism 7.0^35,71^.

## Electronic supplementary material

Supplementary Information

## Data Availability

HCV plasmids, protocols and data will be made available upon request.
