# Supplementary material for: High density Huh7.5 cell hollow fiber bioreactor culture for high-yield production of hepatitis C virus and studies of antivirals
Source: Sci Rep. 2018 Nov 30;8:17505. doi: 10.1038/s41598-018-35010-5 (PMC6269495; doi:10.1038/s41598-018-35010-5)
Supplement: Supplementary file 1 — Supplementary Information [file 41598_2018_35010_MOESM1_ESM.pdf]

High density Huh7.5 cell hollow fiber bioreactor culture for high-yield production of hepatitis C virus and studies of antivirals

Anne F. Pihl[1], Anna F. Offersgaard[1], Christian K. Mathiesen[1], Jannick Prentoe[1], Ulrik Fahnøe[1], Henrik Krarup[2], Jens Bukh[1] & Judith M. Gottwein[1*]

[1] Copenhagen Hepatitis C Program (CO-HEP), Department of Infectious Diseases, Hvidovre Hospital and Department of Immunology and Microbiology, Faculty of Health and Medical Sciences, University of Copenhagen, Copenhagen, Denmark

[2] Section of Molecular Diagnostics, Clinical Biochemistry, Aalborg University Hospital, Aalborg, Denmark

* Correspondence and requests for materials should be addressed to J.M.G. (email: jgottwein@sund.ku.dk)

Supplementary Results

	SA13/JFH1$_{Core-NS5B}$				H77 reference		Sample (allele frequency %)				
Protein	nt pos	nt change	aa pos polyprotein	aa change	aa pos protein	aa pos polyprotein	SA13/JFH1$_{Core-NS5B}$ Inoculum	HCV$_{HFBR}$ Harvest 6	HCV$_{HFBR}$ Harvest 33	sf-HCV$_{HFBR}$ Harvest 11	sf-HCV$_{HFBR}$ Harvest 18
Core	348	C T	3	T M	3	3	-	1.0	16.5	0.8	1.7
	680	C T	114	R W	114	114	99.8	99.8	99.6	99.8	99.8
	900	T C	187	V A	187	187	99.5	98.4	96.0	99.1	99.2
E1	1043	G C	235	V L	44	235	99.7	99.7	99.5	99.8	99.7
	1193	T G	285	F V	94	285	-	1.0	2.9	1.6	3.1
	1290	G A	317	R Q	126	317	-	-	-	0.4	3.6
	1440	A G	367	N S	176	367	-	-	-	1.0	8.9
E2	1493	A C	385	T P	2	385	99.8	99.7	99.6	99.6	99.4
	1529	C A	397	R S	14	397	-	-	-	1.3	7.8
	1937	A G	533	N D	149	532	-	-	1.3	0.7	3.0
p7	2687	T G	783	L V	36	782	99.6	99.6	99.6	99.5	99.7
	2762	G A	808	A T	61	807	-	1.0	4.8	0.8	1.4
NS2	3042	A G	901	Y C	91	900	99.5	99.5	99.4	99.4	99.5
	3405	C G	1022	A G	212	1021	100	100	100	100	100
NS3	3696	A G	1119	K R	92	1118	100	100	100	100	100
	4449	G A	1370	R Q	343	1369	-	0.7	14.5	0.9	1.3
	5088	A G	1583	N S	556	1582	-	-	1.1	1.7	3.1
NS4B	6251	A G	1971	I V	259	1970	-	1.6	4.1	0.8	1.1
NS5A	6443	A G	2035	N D	62	2034	99.7	99.8	99.5	99.8	99.7
	6569	A G	2077	T A	104	2076	0.5	0.8	3.8	2.0	2.5
	6627	A G	2096	Q R	123	2095	-	-	2.2	-	0.4
	7044	A G	2235	E G	266	2238	99.7	99.6	99.4	99.6	99.7
	7086	T C	2249	V A	280	2252	99.7	99.7	99.3	99.5	99.6
	7128	T C	2263	L P	294	2256	99.8	99.7	99.3	99.7	99.8
	7208	T C	2290	Y H	321	2293	-	-	2.3	-	-
	7350	T C	2337	I T	368	2340	99.8	99.9	99.4	99.8	99.7
	7400	T C	2354	S P	385	2357	-	-	-	-	3.0
	7640	G A	2434	D N	441	2413	-	-	2.0	-	-
	7643	A G	2435	T A	442	2414	-	1.9	3.6	-	-
	7646*	A G	2436	T A	445	2417	0.6	4.5	13.9	0.7	0.6
	7647*	C G	2436	T S	445	2417	-	0.8	3.2	-	-
	7649	G A	2437	V M	446	2418	-	1.9	2.4	-	-
	7652	T C	2438	C R	447	2419	0.8	5.0	40.2	-	-
	7652	T A	2438	C S	447	2419	-	2.5	4.4	-	-
NS5B	7658	T A	2440	S T	1	2421	-	1.8	3.6	-	-
	7895	G T	2519	A S	80	2500	99.4	99.2	98.8	99.2	99.2
	7897	A G	2519	A A	80	2500	99.7	99.6	99.4	99.7	99.7
	8919	T C	2860	V A	421	2841	99.7	99.6	99.6	99.8	99.7
**Pairwise distance per site**						π	3.87E-05	1.90E-04	7.30E-04	1.64E-04	4.60E-04
**Pairwise distance per non-synonymous/synonymous site**						πN/πS	0.59	0.60	0.56	0.50	0.42

Supplementary Table S1: NGS analysis of HFBR derived HCV revealed high genetic stability. The HCV second passage stock (SA13/JFH1$_{\text{Core-NS5B}}$ Inoculum; see Materials and Methods) was used for inoculation of both HFBR (Fig. 2 and 3), HCV in harvest 6 and 33 of HFBR cultivated in DMEM+10%FBS (HCV$_{\text{HFBR}}$; Fig. 2) as well as harvest 11 and 18 of HFBR cultivated in serum-free AEM (sf-HCV$_{\text{HFBR}}$; Fig. 3) were subjected to NGS analysis. When a missense mutation occurred at ≥2 % in at least one of the samples, the nucleotide position and the prevalence of the mutation (allele frequency %) in the virus population of all samples are recorded. Nucleotide (nt) and amino acid (aa) positions (pos) are according to the sequence of pSA13/JFH1 (GenBank accession no. FJ393024) and the H77 reference sequence (GenBank accession no AF009606). -, mutation was not detected. Previously described cell culture adaptive mutations are indicated by light grey [1] and dark grey [2] shading and were already present in the HCV second passage stock used for inoculation. *, NGS linkage analysis revealed that mutations in the same codon were not present on the same viral genome. πN/πS, pairwise distance per non-synonymous/synonymous site; π, pairwise distance per site.

**SA13/JFH1$_{\text{Core-NS5B}}$ - NS5A Domain I**					
**SA13/JFH1$_{\text{Core-NS5B}}$**					
**nt pos**	6342	6443	6536	6569	6675
**nt**	T	A	T	A	A
**Bioreactor**					
**HFBR H3**	-	G	-	-	-
**HFBR H10**	-	G	-	-	-
**HFBR H14**	-	G	-	-	-
**HFBR H19**	-	G	-	-	G
**HFBR H22**	-	G	T/C	-	-
**HFBR H26**	T/C	G	T/C	-	-
**HFBR H31**	-	G	C	-	-
**Derived cultures**					
**DC H3**	-	G	-	-	-
**DC H12**	-	G	-	-	-
**DC H15**	-	G	C	-	-
**DC H19**	-	G	C	-	-
**DC H24**		G	C	A/g	
**DC H26**	-	G	C	-	-
**DC H31**	-	G	C	-	-
**aa pos in polyprotein (SA13/JFH1**	2001	2035	2066	2077	2112
**aa pos in polyprotein (H77)**	2000	2034	2065	2076	2111
**aa pos in protein (H77)**	28	62	93	104	139
**aa change**	F-S	N-D	Y-H	T-A	K-R

Supplementary Table S2: Sequence analysis of NS5A domain I revealed resistance mutations in HCV harvested from the HFBR under daclatasvir treatment. Domain I of the NS5A protein of HCV (SA13/JFH1$_{Core\text{-}NS5B}$) was analyzed by Sanger sequencing as described in Materials and Methods in (i) selected HFBR harvests (HFBR Hn, with n indicating the number of the harvest (see Fig. 7)) or (ii) derived cultures (DC Hn, with n indicating the harvest from the HFBR that was used for inoculation of the derived culture). Nucleotide (nt) and amino acid (aa) positions (pos) with changes are shown relative to SA13/JFH1$_{Core\text{-}NS5B.}$ In addition, aa pos are shown relative to the NS5A protein and the polyprotein of H77 (GenBank accession no. AF009606). -, identical with SA13/JFH1$_{Core\text{-}NS5B.}$ The mutation shaded in light grey is a cell culture adaptive mutation already present in the HCV second passage stock inoculum [1]. T/C indicates that the mutation is present as a 50/50 quasispecies. A/g indicates that A is the dominant nucleotide present in the viral population.

Supplementary Materials and Methods

Reverse transcription polymerase chain reaction (RT-PCR) for amplification of HCV sequences. For amplification of HCV NS5A domain I, the High Pure Viral Nucleic Acid Kit (Roche) was used to extract HCV RNA from 200 µL cell culture supernatant. RT-PCR was carried out as indicated in Supplementary Table S3 using primers shown in Supplementary Table S4 and PCR cycling parameters shown in Supplementary Table S5. For amplification of the complete HCV ORF, HCV RNA was extracted from 250 µl supernatant with TRIzol LS and chlorofrom in Gel Lock heavy Eppendorf tubes and purified on RNA Clean & concentrator ™ -5 columns (Zymo Research). RT-PCR was carried out as indicated in Supplementary Table S6 using primers shown in Supplementary Table S7 and PCR cycling parameters shown in Supplementary Table S8.

Supplementary Table S3: Protocol for reverse transcription and, first and second PCR for amplification of NS5A Domain 1

**Protocol for reverse transcription for amplification of NS5A Domain I**	
**Reagent**	**Volume (µL)**
RT primer (10 µM)	2.5
dNTP mix of each dNTP (10 mM)	1
RNA	9
5 min at 65 °C	
5x First Strand Buffer	4
DTT (0.1 M)	1
RNasin	0.5
SuperScript III	2
60 min at 50 °C<br>15 min at 70 °C	
RNase T	1
RNase H	1
20 min at 37 °C	

Protocol for fist PCR for amplification of NS5A Domain I	
**Reagent**	**Volume (µL)**
BD Advantage ™ 2 PCR Buffer	5
dNTP mix of each dNTP (10 mM)	1.3
BD Advantage 2 Polymerase Mix	1
Forward Primer (10 µM)	1
Reverse Primer (10 µM)	1
cDNA	2.5
H_2O	38.2

Protocol for second PCR for amplification of NS5A Domain I	
**Reagent**	**Volume (µL)**
BD Advantage ™ 2 PCR Buffer	5
dNTP mix of each dNTP (10 mM)	1.3
BD Advantage 2 Polymerase Mix	1
Forward Primer (10 µM)	1
Reverse Primer (10 µM)	1
First PCR product	2.5
H_2O	38.2

Supplementary Table S4: Primers for amplification of NS5A Domain 1

Reverse transcription	Primer sequence (5' - 3')
JR7581	GGAGGTTGAAGCTCTACCTG
**First PCR**	**Primer sequence (5' - 3')**
JF5272	TGGCCCAAAGTGGAACAATTTTGG
JR7234	GAAGCTCTACCTGATCAGACTCCA
**Second PCR**	**Primer sequence (5' - 3')**
JF6177	AGCGTGTGACCCAACTACTTG
JR7297	GGGGAGAGCACAACCAGCAAC

Supplementary Table S5: PCR cycle parameters for amplification of NS5A Domain 1

First PCR	Temperature (°C)	Duration
	99	60 seconds
35 cycles	99	35 seconds
	65	40 seconds
	68	3 minutes
	68	2 minutes
	4	∞

Second PCR	Temperature (°C)	Duration
	99	60 seconds
35 cycles	99	35 seconds
	65	60 seconds
	68	60 seconds
	68	60 seconds
	4	∞

Supplementary Table S6: Protocol for reverse transcription and PCR for amplification of the full-length ORF

**Protocol for reverse transcription for amplification of full-length ORF**	
**Reagent**	**Volume (µL)**
RT primer (2 µM)	1
dNTP mix of each dNTP (10 mM)	1
RNasin Plus RNase Inhibitor	1
RNA	12
5 min at 70 °C	
5x RT Buffer	4
Maxima minus H RT	1
RNA-primer mix	15
120 min at 50 °C	
5 min at 85 °C	
RNase H	1
20 min at 37 °C	

**Protocol for PCR amplification of full-length ORF**	
**Reagent**	**Volume (µL)**
5x Q5 Reaction Buffer	10
5x Q5 High GC Enhancer	10
Forward Primer (10 µM)	2.5
Reverse Primer (10 µM)	2.5
dNTP mix of each dNTP (10 mM)	1
Q5 Hot start High-Fidelity DNA Polymerase	0.5
H_2O	21.5
cDNA template	2

Supplementary Table S7: Primers for amplification of full-length ORF

**Reverse transcription**	**Primer sequence (5' - 3')**
J6-JFH1-9472-RT	AGCTATGGAGTGTACCTAGTGT
**PCR**	**Primer sequence (5' - 3')**
JFH1-303-F	CTTGCGAGTGCCCCGGGAGG
JFH1-9467-R	TGGAGTGTACCTAGTGTGTGCCGCTC

Supplementary Table S8: PCR cycle parameters for amplification of full-length ORF

**PCR**	**Temperature (°C)**	**Duration**
	98	30 seconds
35 cycles	98	10 seconds
	65	10 seconds
	72	8 minutes
	72	8 minutes
	4	∞

Supplementary Figures

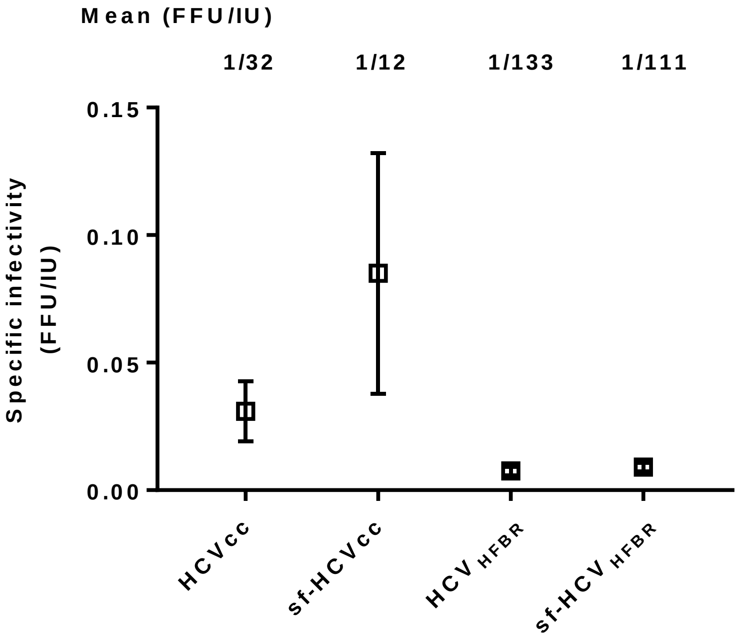

Supplementary Figure S1: Specific infectivity values were calculated for individual cell culture supernatants by dividing HCV infectivity titers (FFU/mL) by HCV RNA titers (IU/mL). Plotted are mean specific infectivity values with standard error of the mean obtained by analysis of multiple cell culture supernatants: For HCVcc and sf-HCVcc, three virus containing supernatants produced for this study were analyzed, respectively. For HCV_{HFBR}, 28 harvests from the bioreactor shown in Fig. 2 were analyzed. For sf-HCV_{HFBR}, 13 harvests from serum-free culture from the bioreactor shown in Fig. 3 were analyzed. On top, mean specific infectivity values are shown for each condition.

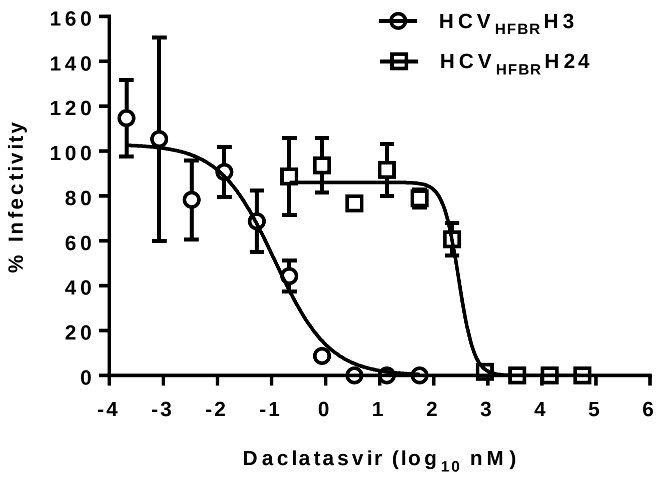

Supplementary Figure S2: HFBR derived escape variants showed resistance to daclatasvir. Supernatant from the derived cultures of HFBR harvest 3 (prior to treatment) and harvest 24 (during treatment) (Fig. 7) was used to infect Huh7.5 cells followed by treatment with daclatasvir at different concentrations as described in Material and Methods. The % infectivity was calculated, and curves were fitted as described in Materials and Methods. Data points are means of three replicates with SEM.

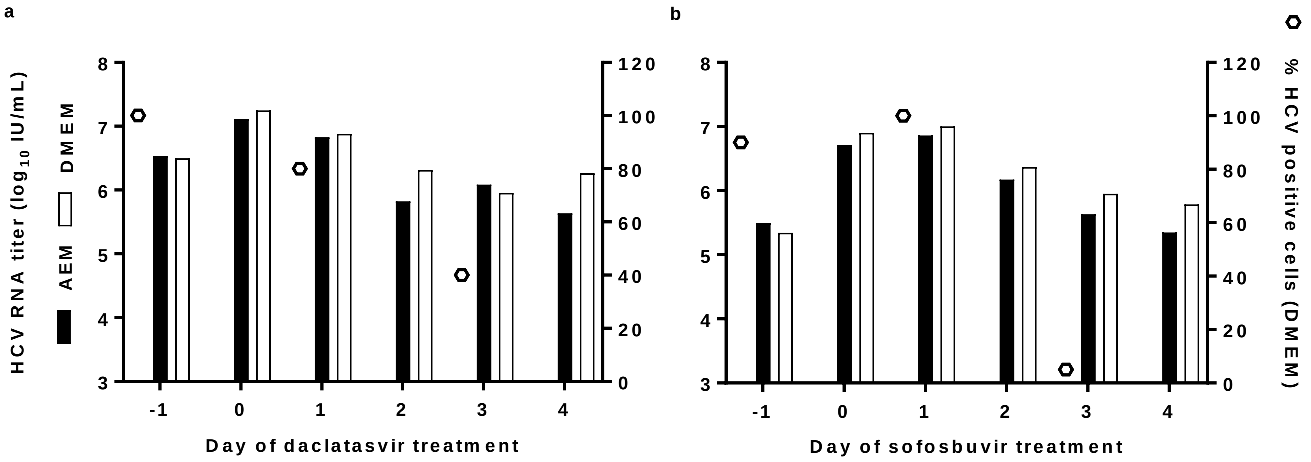

Supplementary Figure S3: Initiation of treatment with daclatasvir or sofosbuvir induced a decline in HCV RNA titers in standard monolayer cell culture. 10^6 Huh7.5 cells plated the previous day in replicate T25 cell culture flasks were infected at an MOI of 0.003 with HCV third passage stock. When virus had spread to >90% of the cells as determined by immunostaining, treatment with daclatasvir at a concentration of 7.8 nM (corresponding to 64 x EC50[3]) or sofosbuvir at a concentration of 5580 nM (corresponding to 10xEC50[4]) was initiated. Replicate cultures in DMEM were split and treated every 2 days; on these days samples for immunostaining were obtained to determine the % of HCV antigen positive cells to monitor viral spread. In replicate cultures, DMEM was replaced by AEM when treatment was initiated. Cells in AEM were treated every 2 days but not split; immunostainings were not done. Supernatants for determination of HCV RNA titers were collected every day.

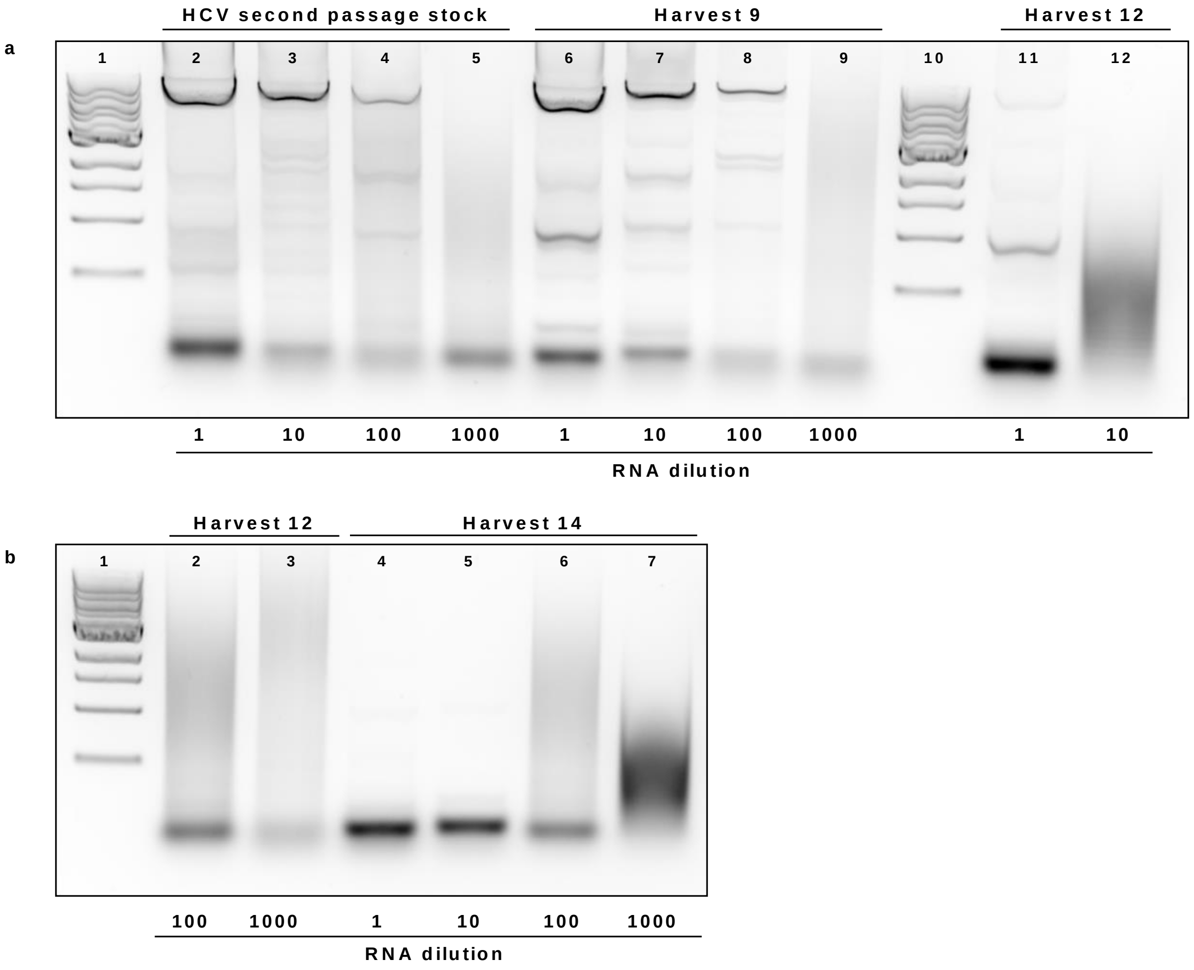

Supplementary Figure S4: Full-length agarose gel for visualization of full-length RT-PCR amplicons spanning the complete HCV ORF. RNA was extracted from a HCV second passage stock serving as positive control as well as from harvest 9, harvest 12 and harvest 14 from the HFBR subjected to treatment with daclatasvir (Fig. 7) and diluted 1-, 10-, 100-, and 1000-fold. Full-length amplicons spanning the complete HCV ORF were generated by RT-PCR. PCR products were visualized on a 1% agarose gel. On the top part of the gel **a)** PCR products derived from the HCV second passage stock were loaded onto lane 2-5, harvest 9 PCR products were loaded onto lane 6-9 and harvest 12 PCR products from 1-, and 10-fold diluted RNA were loaded onto lane 11-12. A 1 kB DNA ladder (New England

Biolabs) was loaded onto lane 1 and 10. On the bottom part of the same gel **b)** harvest 12 PCR products from 100-, and 1000-fold diluted RNA were loaded onto lane 2-3, and harvest 14 PCR products were loaded onto lane 4-7. A 1 kB ladder was loaded onto lane 1. Two images were taken of the top **a)** and bottom **b)** part of the gel, respectively, and cropped for clarity (Fig. 7). Default software (Image Lab 5.2.1, BIO-RAD) settings were used (auto-scale with gamma correction set to *1*).

References

1 Mathiesen, C. K. *et al.* Adaptive Mutations Enhance Assembly and Cell-to-Cell Transmission of a High-Titer Hepatitis C Virus Genotype 5a Core-NS2 JFH1-Based Recombinant. *Journal of Virology* **89**, 7758-7775 (2015).

2 Jensen, T. B. *et al.* Highly Efficient JFHl-Based Cell-Culture System for Hepatitis C Virus Genotype 5a: Failure of Homologous Neutralizing-Antibody Treatment to Control Infection. *The Journal of Infectious Diseases* **198**, 1756-1765 (2008).

3 Gottwein, J. M. *et al.* Efficacy of NS5A Inhibitors Against Hepatitis C Virus Genotypes 1–7 and Escape Variants. *Gastroenterology* **154**, 1435-1448 (2018).

4 Ramirez, S. *et al.* Highly efficient infectious cell culture of three hepatitis C virus genotype 2b strains and sensitivity to lead protease, nonstructural protein 5A, and polymerase inhibitors. *Hepatology* **59**, 395-407 (2013).
